# The Perception of Health Professionals in Bangladesh toward the Digitalization of the Health Sector

**DOI:** 10.3390/ijerph192013695

**Published:** 2022-10-21

**Authors:** Md Shakhawat Hossain, M. M. Mahbubul Syeed, Kaniz Fatema, Mohammad Faisal Uddin

**Affiliations:** 1 Department of CS, American International University-Bangladesh (AIUB), Dhaka 1229, Bangladesh; 2 RIoT Research Center, Independent University, Bangladesh, Dhaka 1229, Bangladesh; 3 Department of CSE, Independent University, Bangladesh (IUB), Dhaka 1229, Bangladesh

**Keywords:** digitalization of healthcare, perceptions, medical education, survey of professionals, medical professionals

## Abstract

Bangladesh is undertaking a major transformation towards digitalization in every sector, and healthcare is no exception. Digitalization of the health sector is expected to improve healthcare services while reducing human effort and ensuring the satisfaction of patients and health professionals. However, for practical and successful digitalization, it is necessary to understand the perceptions of health professionals. Therefore, we conducted a cross-sectional survey in Bangladesh to investigate health professionals’ perceptions in relation to various socio–demographic variables such as age, gender, location, profession and institution. We also evaluated their competencies, as digital health-related competencies are required for digitalization. Additionally, we identified major digitalization challenges. Quantitative survey data were analyzed with Python Pandas, and qualitative data were classified using Valence-Aware Dictionary and Sentiment Reasoner (VADER). This study found significant relationships between age $\chi^{2}\left(12,N=701\right)=82.02,p<0.001$; location $\chi^{2}\left(4,N=701\right)=18.78,p<0.001$; and profession $\chi^{2}\left(16,N=701\right)=71.02,p<0.001$; with technical competency. These variables also have similar influences on psychological competency. According to VADER, 88.1% (583/701) of respondents have a positive outlook toward digitalization. The internal consistency of the survey was confirmed by Cronbach’s alpha score (0.746). This study assisted in developing a better understanding of how professionals perceive digitalization, categorizes professionals based on competency, and prioritizes the major digitalization challenges.

## 1. Introduction

Bangladesh has one of the world’s densest populations, with approximately 21.8% of the population living in poverty and healthcare that lacks reliability [1,2]. Healthcare services have been repeatedly proven insufficient to provide proper support to citizens, especially during emergencies such as the COVID-19 pandemic and dengue epidemic [3,4,5]. The World Health Organization (WHO) recommends at least one doctor for every 1000 patients; however, in Bangladesh, each doctor is responsible for at least 1901 patients, which is the second-lowest ratio in South Asia [6]. According to a survey by the Bangladesh Medical and Dental Council, there were 54,167 registered doctors in 2018; 25,739 were male and 28,425 female [7]. However, Bangladesh has a population of around 162.7 million according to the Bangladesh Bureau of Statistics in 2017 [8]. Thus, patients have to battle to maintain their basic health. In a public hospital outpatient department, a patient has to wait 1.5 h on average to see a doctor for a consultation that lasts less than a minute [9]. There are occasions when even a single doctor is not available. This situation becomes more painful during a crisis, such as COVID or the dengue epidemic, which are common in Bangladesh. According to a survey, almost 80% of people often seek healthcare services from drug stores and are treated by unqualified and unlicensed doctors in Bangladesh [10].

There are problems in diagnostic pathology and radiology as well. There is a scarcity of pathologists and radiologists. Moreover, efficient pathological resources are not available in all hospitals. The number of pathologists is not sufficient, plus it takes a long time to produce skilled pathologists. The assistance provided by technicians, midwives and nurses is also minimal. Akter et al. identified heavy workloads and insufficient technical support as the main problems that affect health workers’ ability to provide care [11]. Furthermore, medical facilities are mainly available in city areas, resulting in a healthcare divide that deprives people living outside the city, where approximately 70% of the health centers do not have the basic equipment necessary for health examinations [6]. Digitalization of the healthcare sector will enable information technology, artificial intelligence (AI) and computer-aided diagnosis (CAD) for healthcare, which are expected to reduce the turnaround times for dealing with patients, medication errors and workload. This will allow pathologists, radiologists and other doctors to deal with a higher number of patients in a shorter time. On the other side, digitalization is also aimed at increasing patient and health professional satisfaction by improving resource allocation, advancing preventive care and ensuring primary care for all [12,13,14].

Digitalization of the health sector is certainly the solution for improving the quality of healthcare, ensuring health equity and increasing productivity and satisfaction of healthcare professionals and patients. Digitalization refers to the complete transformation of the health sector to enable digital health, which includes digital pathology, digital radiology, digital patient management and digital health records under the same umbrella enabled by data-driven information technology and machine intelligence. Its goal is to prevent health issues by enabling early detection of diseases and prolonging life by ensuring effective healthcare through the coordinated efforts of healthcare workers and consumers [15]. On the contrary, digitization is only limited to making a particular action or service digital. Digitization of different health services has been started already in Bangladesh, and some benefits are noticeable [6]. However, to take full advantage of digital health, complete digitalization is necessary.

Multiple studies have reported on and come up with theoretical definitions of digital health; these studies suggest digitalization of healthcare as a solution to improve healthcare quality, ensure equity, reduce costs, labor and complexity, and increase efficiency [16,17,18]. Several countries, including the United States, Germany, and Australia, have started digitization of the health-sector. The digitization of health services has been started in Bangladesh, but it has been slowed down recently. Consequently, the entire digitalization effort has become stagnated. Inadequate digital health literacy and competency have been identified by several studies as the main obstacles to the digitization of healthcare in countries that have initiated the transformation [19,20,21]. Health professionals are the key players in the digital health system, and digitalization will change their roles and duties in an unprecedented way. Therefore, it is necessary to investigate and comprehend their perceptions toward this transformation to make the digitalization of the health-sector successful.

In this paper, we present the findings of a survey of healthcare professionals designed to gain a better understanding of what health professionals think about healthcare digitalization. Additionally, we assess their technical competencies and identify the challenges of digitalization based on the survey responses. We also look into how age, gender, experience level and work location influenced their opinions. This study also gathered data on professionals’ knowledge of digital healthcare, CAD, AI, digital health applications, whole-slide images and other aspects of digital health literacy. Further, we investigated health professionals’ confidence in digitized health systems.

This paper presents useful facts and statistics about health professionals’ familiarity with digital health literacy and confidence in using digital health systems, their preferences, and the limitations of the existing medical curriculum. This will help us better understand the perspectives of health professionals. Furthermore, we believe that the knowledge and insights gained from this study will contribute to the digitalization of healthcare.

## 2. Related Work

The transition from the third industrial revolution to the fourth industrial revolution has intensified the use of technologies such as AI, big-data analysis, Internet of Things (IoT), drones, genome editing, augmented reality, cloud computing and autonomous robots, which are changing how healthcare is delivered. As a result, global efforts to digitalize healthcare have begun. Several studies have investigated the attitudes of healthcare workforces worldwide and have mostly included students and doctors. Most studies that have investigated the attitudes of students toward AI and digital healthcare-based applications, tools and systems reported the positive mindset of students [20,22,23,24,25,26,27,28,29,30]. However, in some studies, students were found to be pessimistic toward the digitization of health services [22,23].

A large number of studies have been conducted in Europe, with Germany leading the way. Multiples studies have investigated the sentiment of German health professionals. Adrian et al. conducted a survey to investigate the attitudes of German medical students towards AI and other digital health applications [20]. Heiko et al. conducted another survey on German medical students to assess their preparation for digitalized medicine [31]. The survey included 453 students and found only 22% (100/453) of them were prepared for the digital transformation, which can be associated with another finding of this study: 70% (318/453) of these students had not participated in any university courses on digital health. The study identified losing patient contact, data protection and medicolegal issues as major concerns of students. However, the students who participated in the study did not express any fear of losing their jobs due to digitization in their future careers. This survey also highlighted the incompatibility of German medical curricular for preparing students for digitalization. Felix et al. [32] conducted another study to identify the emotion of medical students. In their study, they considered students from all over Europe and assessed their knowledge and attitudes toward digital health. This study included 451 students from 39 European countries and found that the majority of them had a positive attitude. However, this study discovered a lack of knowledge and preparedness among students to actively participate in the transformation process. For data analysis, they used R and MAXQDA software.

Maximilian et al. studied the perceptions of German medical faculties towards the digitization of health services [33]. They also assessed the capability of the current medical curriculum and strategies for medical faculties to prepare students for digitalization. Their study reported that the current educational system is mostly failing to prepare students for the transformation, and that the role of the medical faculties requires an update. At the Fraunhofer Institute for Software and Systems Engineering, another survey was carried out to investigate the role of doctors in health digitalization [34]. This study gathered responses from 1274 doctors of various ages from public, private and non-profit health institutions. The survey included a diverse group of German doctors who expressed a strong affinity for digitalization, with 57% actively using digital health-related applications. However, the study discovered that doctors prefer to play a passive role in the digitalization process, such as scrutinizing, which contradicts the theoretical explanation of digitalization. This indicates the importance of proper display and adequate training on digital health, in which the roles of health professionals will be well-defined for the digital transition.

Outside of Germany, Anna Wernhart et al. [35] conducted an online survey in Austria to determine medical students’ and other medical employees’ perceptions of eHealth and telemedicine. This study reported a lack of familiarity, particularly among employees. Sayed et al. [36] conducted a survey in five regions of Saudi Arabia and identified privacy, staff compliance and cost as the major barriers to the adoption of digital health records in dental healthcare. Johanna Tolonen et al. [37] surveyed Finnish medical students to assess their competencies in using information technology for health services and found the existing education system inefficient. A limited number of surveys have reported the digitization and digitalization of health in Bangladesh. Islam et al. investigated the usability of specific mobile health applications (mHealth) in Bangladesh [38]. Their findings indicate that the usability of Bangladesh’s current mHealth applications is inadequate. However, they only collected responses from 50 subjects, including medical students, doctors, bankers and other service providers. As a result, generalizing their findings is extremely difficult. Rana et al. conducted another survey to investigate students’ attitudes toward digitalization [39]. However, in their survey, they considered the digitalization of all sectors rather than only health, and they targeted students from public and private schools, colleges and universities. In total, 60 students took part in this survey and expressed support for digitalization, specially in the communication, health and education sectors. Khan et al. conducted another study to identify the impressions of Bangladeshi people about digital healthcare [40]. This study also reported a positive attitude of Bangladeshi citizens towards the digitalization of healthcare.

The lack of sufficient research to understand the perceptions and competencies of the health workforce have contributed to the stagnation of healthcare digitalization in Bangladesh. This necessitates research of healthcare professionals to understand their feelings and preparedness for the transformation. As a result, we surveyed Bangladeshi healthcare professionals to ascertain their perceptions and assess their technical competency and psychological preparedness for the digitalization of the health sector. This research also seeks to identify the major challenges of healthcare digitization.

## 3. Methods

### 3.1. Survey Design

We conducted a descriptive cross-sectional study to understand health professionals’ perceptions of healthcare digitalization, in which the roles of various socio–demographic variables such as age, gender, location, profession and institution were investigated. We also investigated the role of technical competency in influencing perception.

This survey was carried out in collaboration with the public health departments of Independent University, Bangladesh, and American International University, Bangladesh. From 22 June to 30 August 2022, the survey was available online. For this survey, we assembled a team of experts that included two doctors, two public health professionals and four researchers. Another group of 12 volunteers, comprised of medical, public health and undergraduate students as well as doctors and research assistants, was ready to connect healthcare professions for the survey. Initially, a set of 30 candidate questions was prepared, taking into consideration the objectives of this study and the existing literature. The expert team then scrutinized the candidate questions, which were then ranked by each member to finally develop a set of 16 questions. The criteria for selecting the questions were to meet the primary objectives of the study while keeping the response time under 15 min. The expert committee had 100% agreement on the questionnaire to ensure inter-observer agreement (IOA).

**Figure 1:**
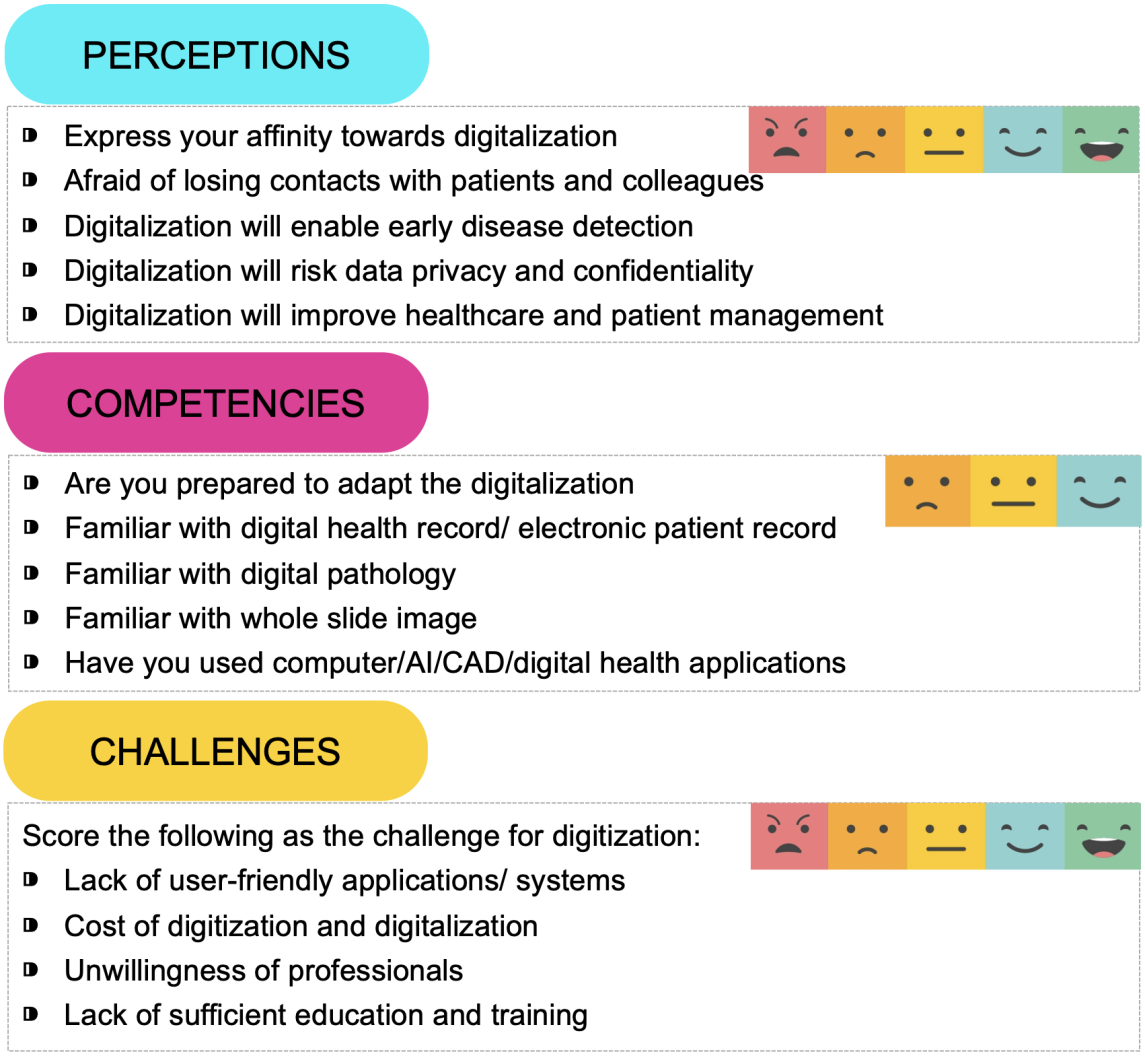
Example of questions designed to gather responses on three different aspects.

Examples of some questions are given in Figure 1. This survey required an attendee to select appropriate options in 38 cases and written answers for two questions. Three types of psychometric response scales were used: the 2-point, 3-point and 5-point Likert scales. The 2-point Likert is binary as (1) No or (2) Yes. The 3-point Likert scale contains the points: (1) Completely unknown; (2) Basically known; and (3) Completely known. The 5-point Likert scale contains: (1) Strongly disagree; (2) Disagree; (3) Neutral; (4) Agree; and (5) Strongly agree. The survey also required participants to write their feelings towards digitalization. In our primary investigation, the maximum response time was found 15 minutes to complete the survey. The survey was conducted in a hybrid mode, with some respondents taking the survey online and others filling out a printed questionnaire with the help of a volunteer. In both cases, the respondents remained anonymous.

### 3.2. Data Collection and Surveyee Inclusion Criteria

Firstly, we prepared a list of health institutions, which included all the public medical colleges and hospitals and only those private medical colleges and hospitals that have an active license number. Currently in Bangladesh there are 960 licensed private hospitals and 109 medical colleges offering a Bachelor of Medicine and Bachelor of Surgery (MBBS) degree, which includes 37 public colleges offering 4350 seats and 72 private colleges offering 6336 seats. Thus, each year the country offers 10,686 medical seats to the candidates. This list of health institution was used to invite health professionals to participate in the survey. In our survey, we categorized the health professionals into six groups: medical students, doctors, pathologists, radiologists, technicians and others. A medical student means someone who is enrolled in medical school or doing his/her residency. However, students who had been admitted but had not started school were excluded from this survey. Pathologists are those who have a Doctor of Medicine (MD) in Pathology and are actively working as a pathologist. Radiologists are those doctors who are specialized in radiology and are currently working as a radiologist. The rest of the doctors, such as cardiologists, dermatologists, neurologists and oncologists, were termed ‘doctors’ in this survey. We wanted to study the attitudes of pathologists and radiologists separately, as they play crucial roles in the digitalization process. As a result, they were categorized differently. The rest of the physicians, on the other hand, were assigned under the ‘doctor’ category, which is a limitation of this survey, but such categorization was done to reduce complexity. Technicians are those who assist in using medical systems. We also considered nurses and midwives in our survey, but they fall under the ‘other’ category.

We tried to include health professionals from all sectors in this survey, including city, suburban and rural areas, public and private hospitals, and new and experienced professionals. The survey was conducted over the internet as well as through a physical meeting, mostly in rural areas. Approximately 14% (100/701) of the survey was conducted in person using a printed questionnaire. All survey respondents gave their permission for their responses to be used for research and publication while remaining anonymous.

### 3.3. Data Curation

The data were curated in two phases: (1) data elimination and (2) data correction. Firstly, incomplete or ill-suited responses were eliminated. As a result, among the 738 respondents, 21 respondents were eliminated due to their ill-suited occupations, and another 15 respondents were eliminated as their responses were incomplete. Finally, 701 respondents were selected after the data-elimination phase, representing roughly 95% (701/738) of the original respondents. After data elimination, we corrected the data inconsistencies. One of the most significant inconsistencies was the name of the institution or organization. We gave each institution a unique name. Another issue concerned the professions; we changed orthopedic surgeons and oncologists to doctors.

### 3.4. Data Analysis Tools

For data analysis, we mainly used Python’s Pandas library, which supports tabular data manipulation using dataframes. The Matplotlib, Seaborn, Ggplot and Plotly libraries of Python were used for data visualization. We also used the NumPy library, which supports multidimensional arrays. Statistical analyses such as the measure of central tendency and dispersion, Chi-Square test, Student’s *t*-test and Pearson’s correlation analysis were performed using the statistical function and data analysis toolpack of MS Excel.

**Figure 2:**
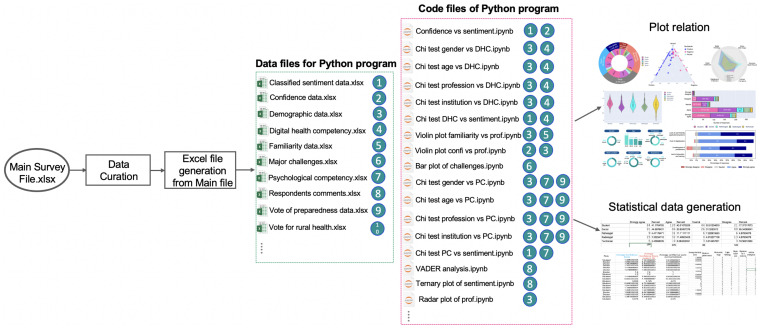
Overview of data analysis.

Figure 2 shows the overall process of data analysis. At first, data curation was applied to the main data file containing the survey responses. The corrected data file was then split into multiple excel/CSV files for importing data into the specific Python program files; then, Python codes were developed to analyze the data and generate graphs. Respondents of this survey were asked to express their feelings about healthcare digitalization in a few sentences, which were then analyzed using sentiment analysis. VADER [41] is a popular sentiment analyzer for classifying sentiments from natural languages, particularly on social media platforms such as Facebook and Twitter. We used a pre-trained VADER [41] model that is available in the Python SentimentIntensityAnalyzer library to classify respondent’s comment as positive, negative or neutral.

## 4. Results

The main outcomes of the survey were the attitudes of health professionals toward digitalization, assessment of their technical and psychological competencies, and perceived digitalization hindrances. The first aspect was assessed through the Likert-scale responses to multiple-choice questions and sentiment analysis of their comments on digitalization. Technical competency was assessed based on familiarity with digital health literacy and previous experience with digital technologies. Psychological competency, on the other hand, was estimated based on their confidence in digital health systems and self-assessment of adaptability for the transition to digital health. Challenges were identified based on participants’ comments, responses to multiple-choice questions and review of the existing literature. We also evaluated the internal consistency of the survey. The Cronbach’s alpha value was estimated for the relevant questions that should have similar responses for the same respondents. The Cronbach’s alpha value for the five-option Likert scale was 0.746, which ensures the consistency of the survey. Section 4.1 shows the statistics of the responders. The data analysis results of the survey are presented in three subsections related to the objectives of the study, given in Section 4.2, Section 4.3, Section 4.5.

### 4.1. Distribution of the Respondents

The total population of healthcare professionals practicing in Bangladesh is unknown and large. Therefore, a reasonable sampling size for this survey was estimated using Equation (1):

(1) $$Sample\;Size=\frac{Z^{2}*S\left(1-S\right)}{e^{2}}\;$$

where *Z*, *e* and *S* are the Z-score, margin of error and standard deviation, respectively. The sample size was estimated with a 90% confidence level, 50% standard deviation and a 3% marginal error. For 90% confidence, the Z-score, marginal error and standard deviation would be 1.65, 0.03 and 0.5, respectively, which would require a necessary sample of approximately 756. In this study, we collected data from 738 respondents initially and then selected 701 respondents after data curation. We attempted to include respondents from both public and private universities, rural and urban areas, and both men and women when conducting the survey.

**Figure 3:**
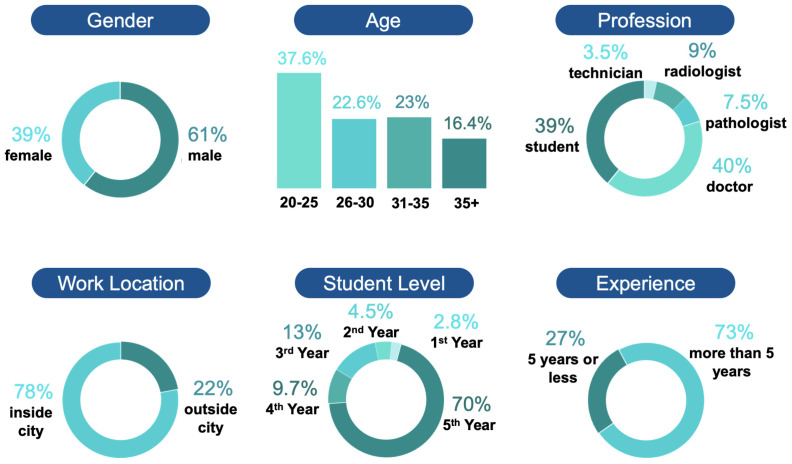
Demographic details of the respondents.

This survey included participants from all (37/37) public medical colleges and hospitals, 45 (45/72) private medical colleges and 33 (33/960) private hospitals and health centers. The respondents were aged between 20 to 65 years, with 431 men (61.48%) and 270 women (38.51%). The largest proportion of respondents 40% (286/701) was doctors, followed by students 39% (274/701). The rest included 53 pathologists, 64 radiologists and 24 technicians. The respondents were mostly from cities (546/701) and had a considerable amount of experience. Approximately 77.7% (332/701) of non-student professionals had more than 5 years of experience. Figure 3 depicts the demographic data of the respondents.

### 4.2. Perception of Professionals

We investigated health professionals’ perspectives on various aspects of healthcare digitalization, such as the role of digital healthcare in improving rural health services, fear of losing skills and jobs, fear of losing contact with colleagues and patients, and data security as a result of digitalization. We also depicted their proclivity for digitalization and their assessment of how well they are prepared for the change.

**Figure 4:**
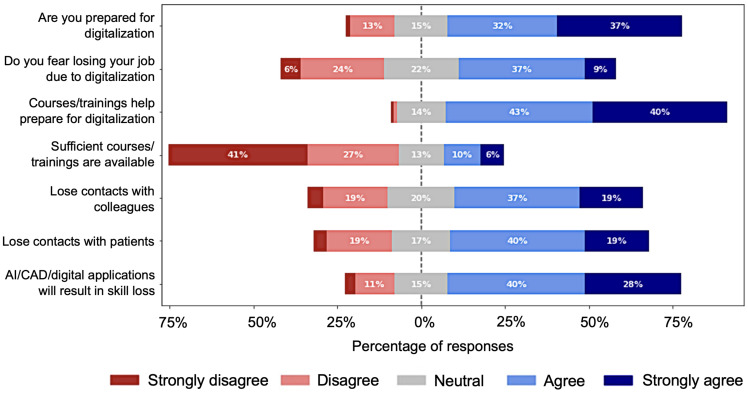
Impressions of professionals on different aspects of digitalization.

**Figure 5:**
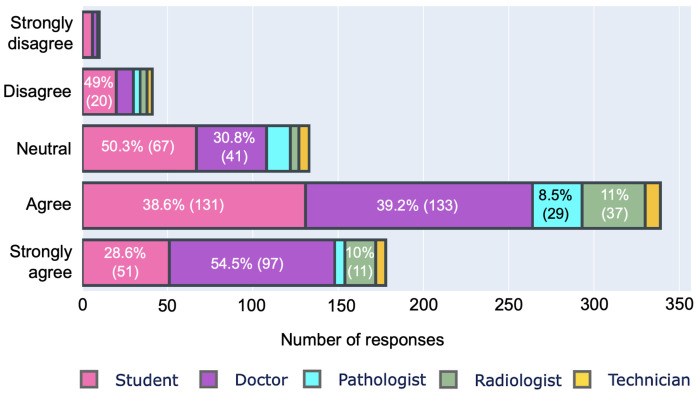
Data privacy and confidentiality risk in digitalization.

Figure 4 depicts the responses for the major concerns of health professionals regarding digitalization, revealing that the majority of the respondents (323/701) fear losing their jobs as a result of this transformation in the future in comparison to the 210 professionals who did not express such fear, while approximately 22% (167/701) of professionals were unsure about it. Similarly, the majority of respondents 68% (477/701), believe that AI, CAD and/or other digital health applications and systems will result in skill loss. The majority also believe that digitalization will reduce communication between health professionals and patients. When asked to rate their readiness for digitalization, 69% (484/701) said they are prepared, while only 13% (92/701) think they are not prepared. The majority of the respondents, approximately 83% (582/701), agree to varying degrees that appropriate training and courses help prepare for digitalization. They are, however, dissatisfied with the available training and courses. Only 16% (113/701) of respondents believe available courses and training are adequate. In terms of data security in digital healthcare, 73.75% (517/701) of professionals believe that digitization will jeopardize data privacy and confidentiality, as shown in Figure 5. Only 7.27% (51/701) of respondents denied this fact.

**Figure 6:**
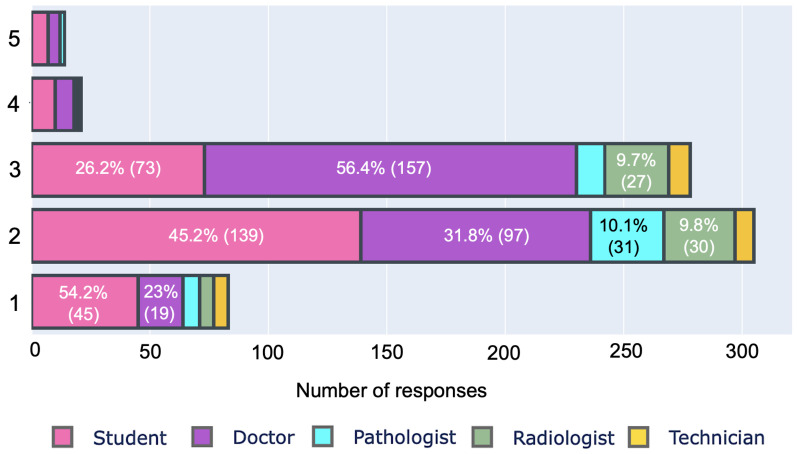
Rating of outside-city healthcare service on a scale of 1–5, if 5 is the city healthcare.

**Figure 7:**
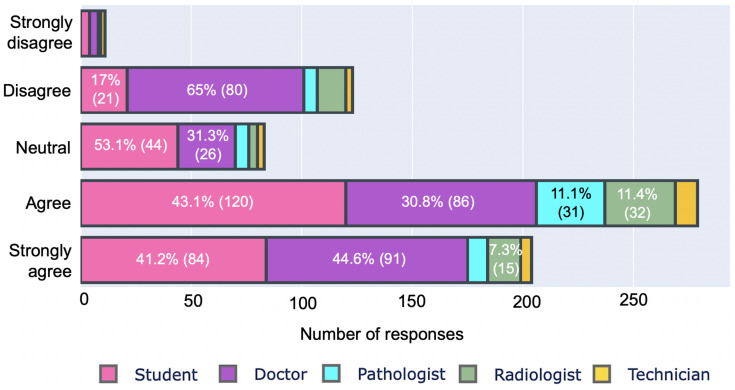
Digitalization for improving healthcare services of rural and remote areas.

We also investigated their concerns about the quality of health services in rural areas and the role of digitalization in improving the situation, as shown in Figure 6 and Figure 7, respectively. We asked respondents to rate rural health services on a scale of 1 to 5, with 5 representing inside-city health services. A total of 95% (666/701) of respondents rated rural health below 4, with 11.8 % (83/701) giving 1 to rural health. In terms of the role of digital health, 68.90% (483/701) of respondents believe that it will improve rural health services and ensure equity. On the contrary, 19% (134/701) of professionals disagreed with this statement. Professionals who rejected digitalization as a means of improving rural healthcare are mostly doctors (approximately 65% (80/123)). On the other hand, 74.4% (204/274) of students trusted digitalization.

**Figure 8:**
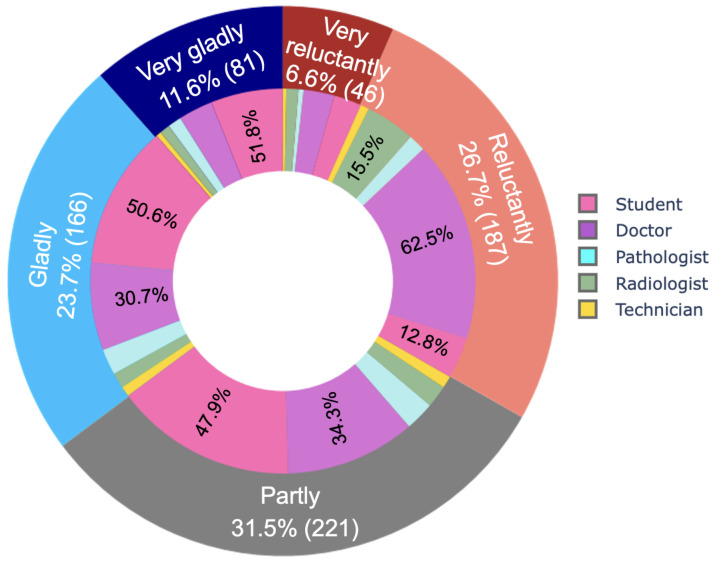
Affinity towards digitalization.

Finally, we asked respondents to express their interest in transitioning to digital health on a 5-point Likert scale to better understand their affinity for healthcare digitalization. Figure 8 reveals that in total, 33.3% (233/701) of respondents did not want the transformation, compared to 35.3% (247/701) who had a strong preference for digitalization. Another 31.5% (221/701) of respondents desired the transformation in part. Doctors make up the majority of those who were reluctant about digitalization (approximately 62.5% (117/187)). Unlike previous research, which has mostly reported a positive attitude toward digitalization for all types of health professionals, this study found a mixed attitude, where students are mostly found positive and confident.

#### Sentiment Analysis of Respondents’ Comments

**Figure 9:**
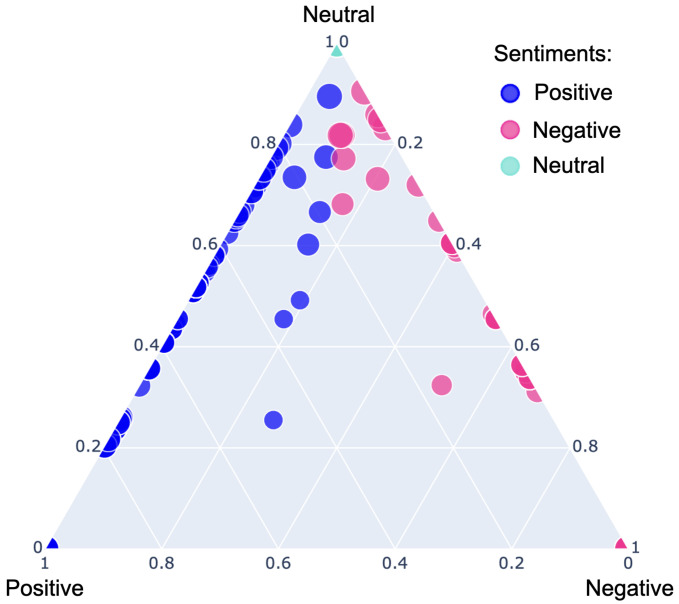
Sentiment classification of surveyees using VADER.

**Figure 10:**
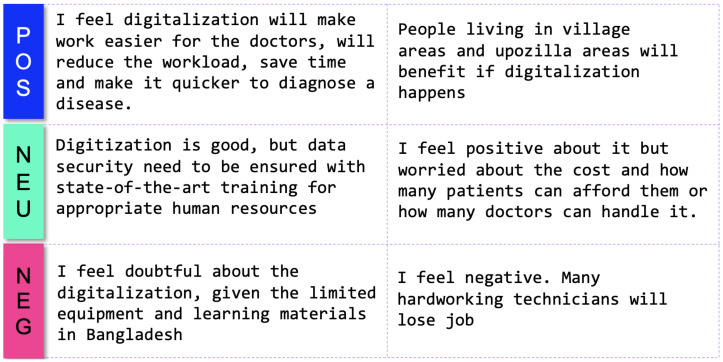
Example of comments and their class predicted by VADER.

The survey questionnaire also included a text box allowing the responders to write their feelings about health digitalization. We classified the comments as positive, negative or neutral using VADER [41]. VADER is a popular unsupervised sentiment analysis method. It can detect emotions as well as the intensity of emotion in words. It assigns a score to each word based on its emotion and then estimates the sentiment of the sentence based on the total emotion score. Then, the score is then normalized from −1 (most extreme negative) to 1 (most extreme positive) for classifying the sentence’s sentiment using Equation (2). Positive emotion is expressed by words such as love, enjoy, happy, like, confident, positive and interested. Words such as unhappy, doubtful and negative, on the other hand, express negative emotion. VADER is intelligent enough to recognize basic context, such as “did not love” as a negative statement. It is also sensitive to punctuation, case, emojis, degree modifiers, slang, and conjunctions, making it highly adaptable. VADER classified 83.1% (583/701) of comments as positive, 4.85% (34/701) as neutral or missing, and only 11.98% (74/701) as negative, as shown in Figure 9. Figure 10 shows examples of comments and their class predicted by VADER.

(2) $$\begin{array}{cc} Positive: & If\;Compound\;Score\;\geq 0.05,\\ Neutral: & If\;Compound\;Score\;>\;-0.05\;and<0.05,\\ Negative: & If\;Compound\;Score\;\leq 0.05 \end{array}$$

### 4.3. Assessment of Competency

We evaluated the competencies of the professionals in two aspects: (1) technical competency and (2) psychological competency. Technical competency was estimated based on their familiarity with digital health literacy and hands-on skills in digital technology, as explained in Section 4.3.1. On the other hand, psychological competency was estimated based on their confidence in digital health and their response to the preparedness for digitalization, as given in Section 4.3.2. Finally, we categorized the workforce based on their competencies.

#### 4.3.1. Technical Competency Related to Digital Health

We evaluated the hands-on skills of respondents based on their skills and experiences with three things: (1) computers or similar computing devices, (2) digital health-related applications and (3) AI/CAD based systems. Approximately 96% (673/701) of the respondents used computers, while only 37% (260/701) used AI/CAD systems and 39% (274/701) digital health applications such as Patient Aid and/or DIMS. Table 1 shows that only 7.7% (54/701) of professionals have operational experience with all three, and 40 (40/54) of them are based in city areas, while 1.5% (11/701) have no experience with computers or any digital health systems. From Table 1, it can be observed that city professionals have more operational experience with digital healthcare compared to outside-city professionals. However, no such differences were noticed between public and private professionals. Table 1 also suggests that students and doctors have higher exposure to digital health technology compared to pathologists, radiologists and technicians.

**Figure 11:**
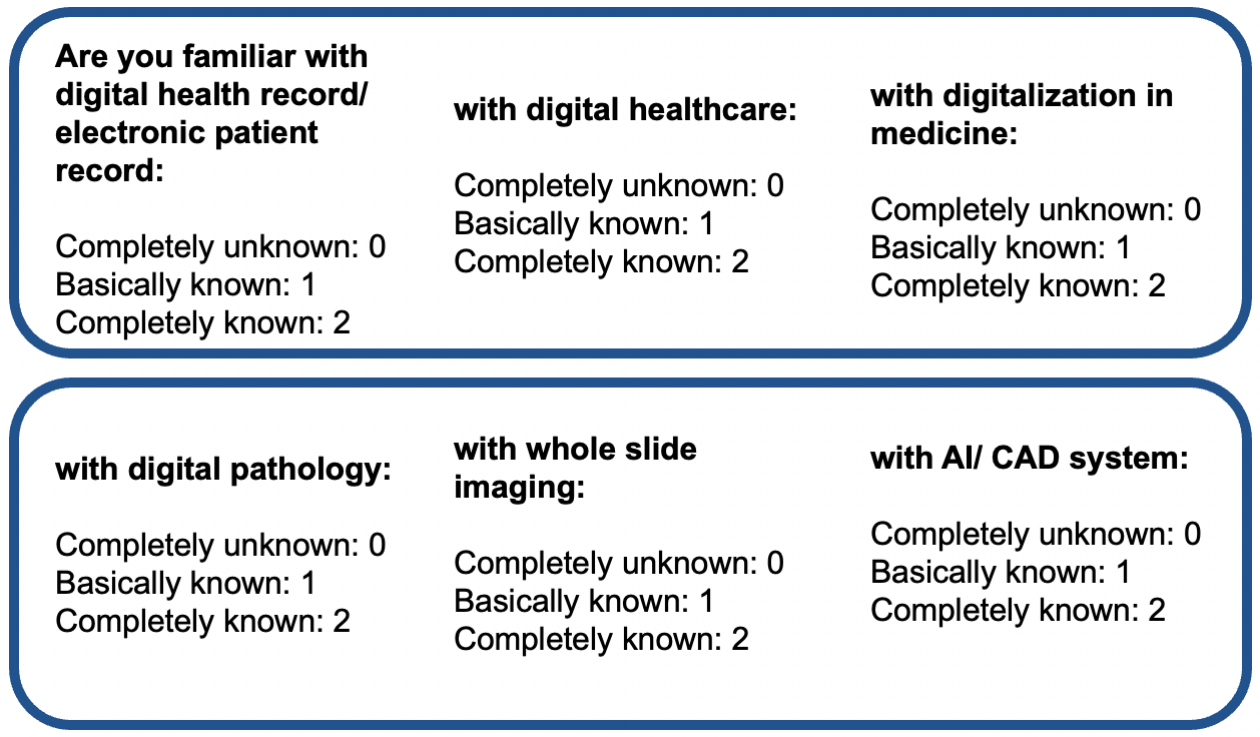
Familiarity scoring system.

**Figure 12:**
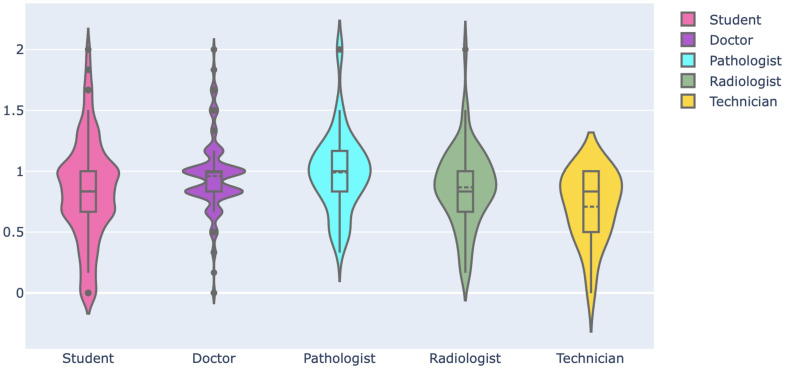
Familiarity score distribution among professionals.

**Figure 13:**
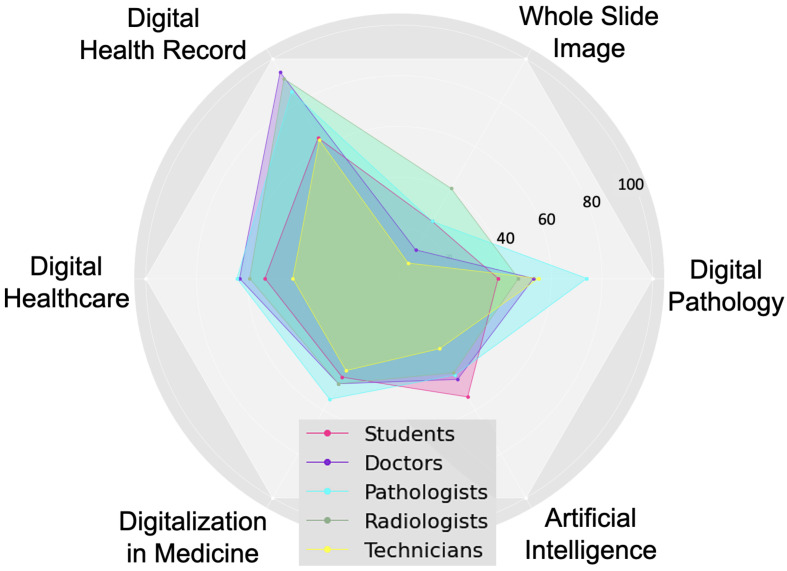
Overall familiarity with digital health literacy-related keywords.

Then, we assessed the familiarity of respondents with digital health literacy. For this, we selected six pertinent keywords and then asked the respondents to rate their familiarity using a 3-point Likert scale, where each option had a numerical score, as shown in Figure 11. The mean and standard deviation of the familiarity score considering all respondents are 0.89 and 0.36, respectively. The familiarity score distribution was compared among professionals using a violin plot, which is useful to visualize multi-modal data distribution, as shown in Figure 12. It shows multiple modes for students and doctors, compared to others that have a single mode. However, the interquartile range (IQR) of doctors is lower than that of the students and others, which means the familiarity scores of doctors mostly center around the mean. Pathologists had the highest mean (mean: 0.99, Std: 0.33), followed by doctors (mean: 0.96, Std: 0.30) and radiologists (mean: 0.86, Std: 0.32). Technicians had the lowest mean of 0.70. The students had a mean of 0.83 and the highest deviation of 0.42. Further, the weighted familiarity scores of professionals for the keywords were compared, as shown in Figure 13. The weighted familiarity score for each keyword was calculated using Equation (3). Then, the weighted score was normalized from 0 to 100. From Figure 13, it can be seen that doctors, pathologists and radiologists have comparatively higher familiarity than students and technicians. All the professionals have low familiarity with whole-slide imaging and high familiarity with digital health records.

(3) $$\begin{array}{c} Weighted\;Familiarity\;Score=2*frequency\;of\;Completely\;Known+\\ 1*frequency\;of\;Basically\;Known+\\ 0*frequency\;of\;Completely\;Unknown \end{array}$$

Then, the familiarity score distribution was compared for different age groups, genders, locations and institutions. The 35+ age group professionals had the highest mean of 0.97, followed by the 31–35 (mean: 0.92, Std: 0.27) and 26–30 (mean: 0.90, Std: 0.33) groups. Male professionals (mean: 0.92, Std: 0.42) had slightly higher familiarity than female (mean: 0.90, Std: 0.36). City professionals had a higher mean and lower standard deviation (mean: 0.92, Std:0.31) compared to those outside cities (mean: 0.87, Std: 0.37). Private professionals (mean: 0.90, Std: 0.32) and public professionals (mean: 0.89, Std: 0.40) had similar distributions.

After that, the hands-on skills in digital technologies and familiarity with digital health literacy were averaged to obtain technical competency. Then, Chi-square tests of independence were performed to examine the relation between technical competency and age, gender, location, profession and institution, as shown in Table 2. No association between gender and institution was found with technical competency, as the Chi-square value was lower than the critical value. However, significant influence was identified for age, location and profession on the technical competency, which resulted in Chi-square values greater than the critical value and p-values less than the alpha significance. Professionals aged 35+ and professionals from city areas showed higher competency. Again, doctors and pathologists showed higher competency compared to others.

#### 4.3.2. Psychological Competency

**Figure 14:**
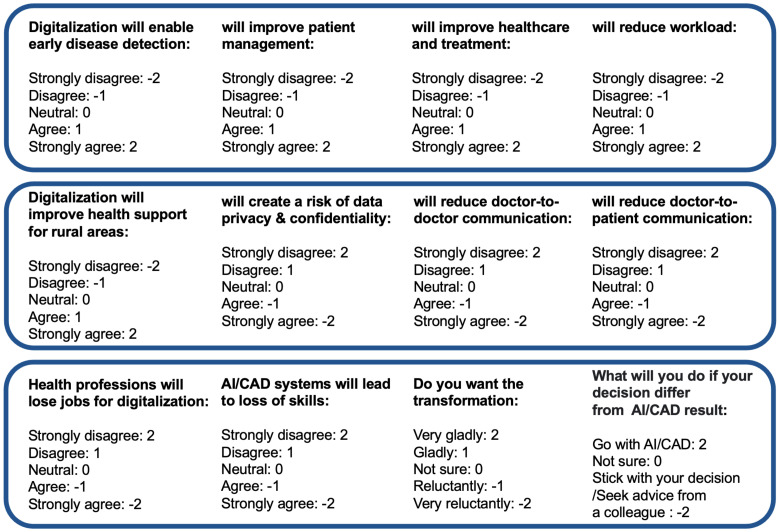
Confidence score calculation.

**Figure 15:**
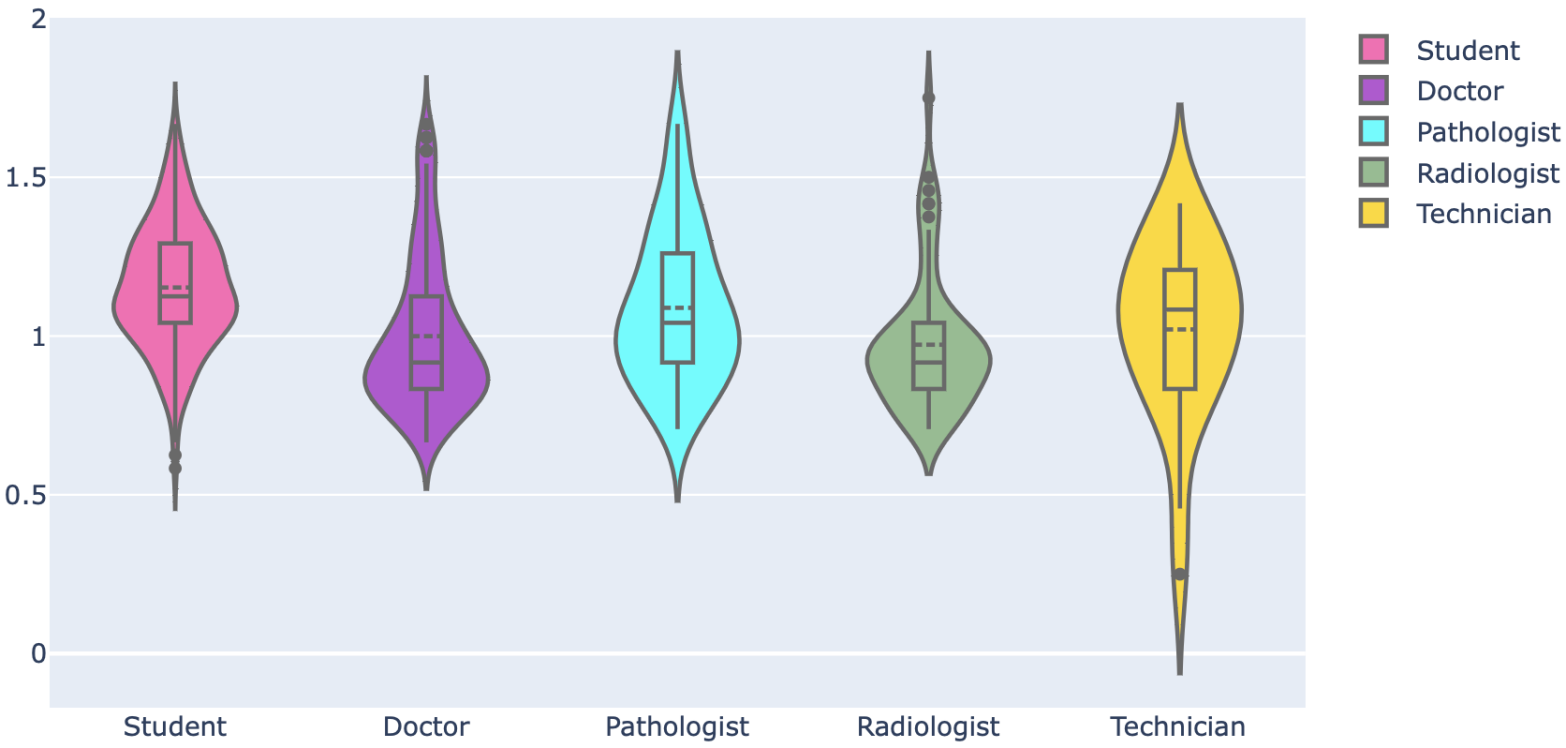
Average confidence score distribution among the professionals.

The psychological competency of professionals was estimated based on their vote of confidence in the digital health system and their self-assessment of preparedness for the transition. We checked the distribution of confidence scores across the professionals. Figure 14 shows how the responses were converted to a numerical score to estimate the average confidence score for a respondent. Then, this score is normalized from 0 to 2. The mean confidence score was 1.06, and the standard deviation was 0.23. The distribution of scores for professionals is shown in Figure 15. All the distributions show a single mode and a narrow IQR with a mean close to 1, which indicates high confidence and less divergence among the professionals. The students had the highest mean of 1.15 and the lowest standard deviation of 0.19. The mean was lowest for the pathologists at 0.97. Then, we investigated the influence of age, gender, location and institution on the confidence score. The 20–25 age-group professionals had the highest mean of 1.13, followed by the 26–30 age group (mean: 1.09, Std: 0.25). The 31-35 (mean: 0.98, Std: 0.22) and 35+ (mean: 0.99, Std: 0.23) age groups had similar distribution. The male (mean: 1.09, Std: 0.23) and female (mean: 1.07, Std: 0.23) professionals had no significant differences as well. Similarly, public (mean: 1.05, Std: 0.24) and private (mean: 1.06, Std: 0.22) professionals had comparable distributions. The city professionals had a slightly higher mean of 1.09 than those outside cities (mean: 1.05).

After that, the confidence score and the self-assessment score of preparedness were averaged to estimate psychological competency. We performed Chi-square tests of independence to examine the relations between psychological competency and age, gender, location, profession and institution, as shown in Table 3. The Chi-square values were lower than the critical values for gender and institution, which indicates no relation for these variables with psychological competency. However, significant influence was identified for age, location and profession, which resulted in Chi-square values greater than the critical value and *p*-values less than the alpha significance. Students and professionals aged 20–25 showed higher psychological competency compared to others.

### 4.4. Identification of Potential Workforce

**Figure 16:**
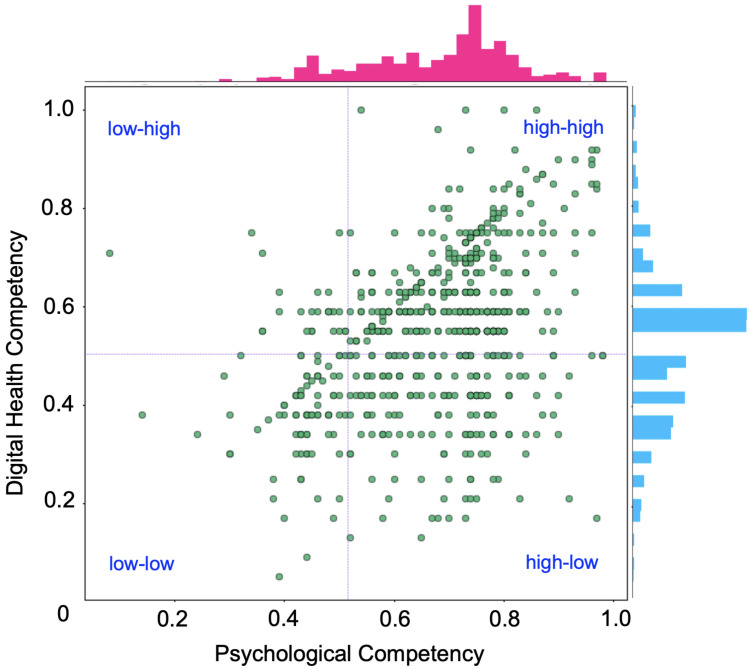
Identification of potential workforce for healthcare digitalization.

We utilized psychological competency and technical competency to identify the potential workforce for digitalization. For this, we have normalized the both scores from 0 to 1 and plotted them in a scatter graph, as shown in Figure 16. This bi-variate analysis indicates low correlation between psychological competency and technical competency, which could have been caused by the technician and student groups, who had low digital competency compared to psychological competency. The Pearson correlation coefficient was 0.40 for all respondents, which climbed to 0.70 if students and technicians were excluded, indicating a positive correlation between psychological and digital competency.

Further, we divided the scatter graph into four regions to categorize the potential of professionals. For example, in the low–high region, respondents have low psychological competency but high digital competency. The high–high region professionals have the most potential for digitalization. This model can be used for designing training or courses and preparing professionals for digitalization. For example, respondents in the low–low region require both courses to boost their mindset and training to improve competency with digital technology.

### 4.5. Major Challenges of Digitalization

**Figure 17:**
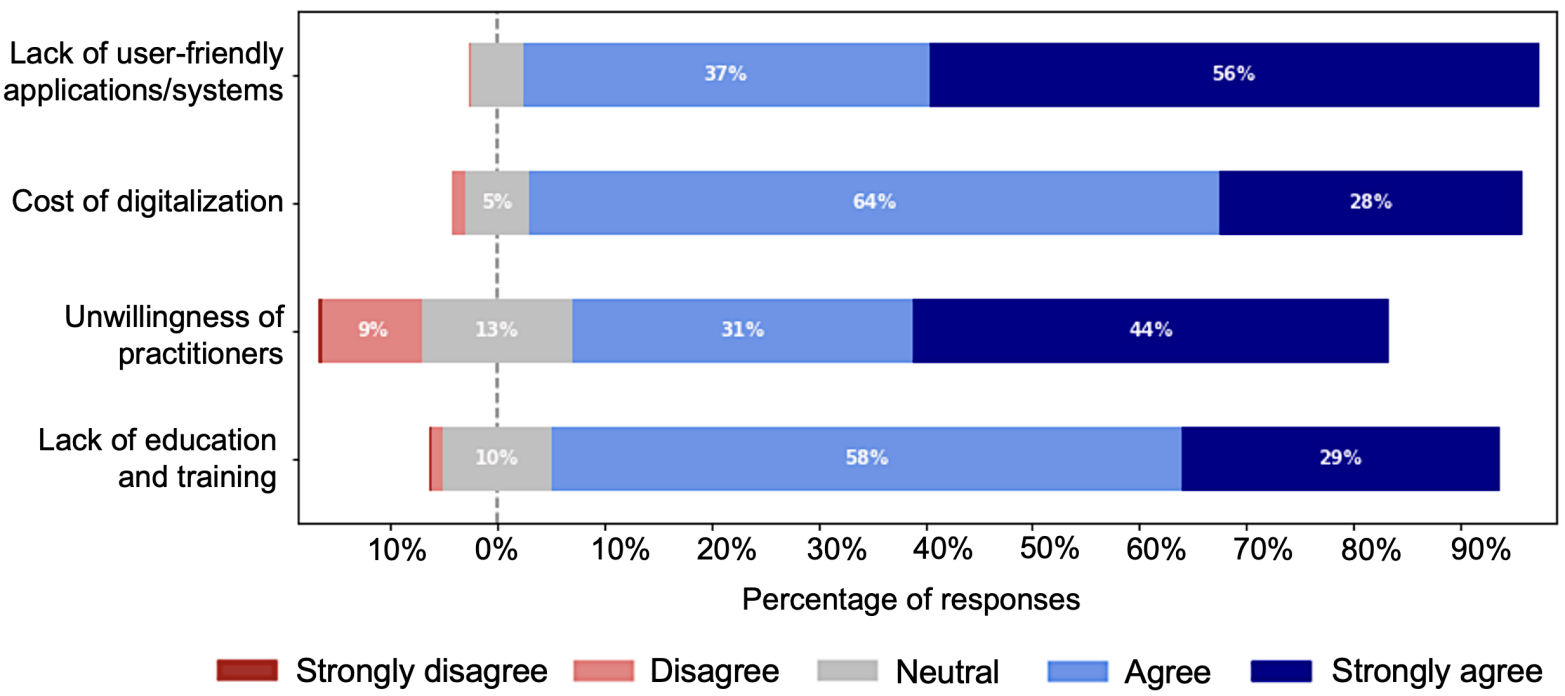
Survey responses on the major challenges for digitalization.

We asked the respondents to rate four pre-selected challenges using the 5-point Likert scale, as shown in Figure 17. The challenges were selected and included in the survey based on voting by experts. From the figure, it is clear that more than 85% of the total surveyees acknowledged these as major challenges.

#### 4.5.1. Lack of User-Friendly Applications and Lack of Will of Professionals

Approximately 94% (665/701) of the respondents identified the lack of user-friendly applications or systems as a major problem. Among the 665 respondents, 640 (96%) have used computers or similar smart devices, 261 (39%) have used AI/CAD systems, and 256 (38%) of professionals have used digital health-related applications. This signifies the limitations of existing healthcare applications. Around, 75% (535/701) recognized the unwillingness of professionals as another major problem, which included 33% (177/535) of student and 66% (358/535) of other professionals. These 358 professionals included 67% (242/358) of doctors, most (57% (205/358)) belonged to the private sector, and 73.5% (178/242) had more than 5 years of experience. Based on the percentage of strong agreement, we can consider lack of user-friendly applications and unwillingness of practitioners as the top two challenges.

#### 4.5.2. Lack of Adequate Training and Courses

Lack of education and training was considered a challenge by 87% (610/701) of respondents. Approximately, 96% (275/286) of doctors thought the education and training for preparing health professionals for digitalization were not sufficient, while 94% (258/274) of the total students agreed with that fact; among them, 39% (107/274) gave strong agreement, and 55% (151/274) gave usual agreement.

#### 4.5.3. Quality of Medical Education and Training

Lack of confidence in AI/CAD and digital health applications, fear of losing job and skills, fear of losing colleagues and patients contacts, lack of preparation, and misconception that digitalization will only benefit city people all signify the lack of an efficient medical curriculum and strategy for preparing healthcare professionals. Therefore, we investigated the medical curriculum of Bangladesh and compared it with the curriculum of other countries. In order to investigate the curriculum, experts selected 10 keywords related to digital health literacy, and we counted their frequency in the medical school syllabi of four different countries, as shown in Table 4. This investigation gives a rough estimate of how well the medical curriculum is designed to prepare the student for digitalization.

It seems the Johns Hopkins University School of Medicine, USA, is more organized to reflect digitalization compared to the other universities. However, for the Medical School of Tokyo University, Japan, we translated the Japanese version of their syllabus to English and then searched for the keywords. In some cases, the translation was not appropriate, which is one of the limitations of this investigation. The scopes of improvement in the Bangladesh Medical and Dental Education curriculum for adapting digitalization can be easily identified from the table. These are few or no mentions of terms such as machine learning, artificial or machine intelligence, informatics or whole-slide image. This signifies the limitations of the medical curriculum in Bangladesh for preparing students for digitalization.

Furthermore, a 65% shortage of teaching staff has been reported in both public and private medical institutions in Bangladesh [42]. Thus, students lack proper practical training and expertise. In general, medical education should include 80% practical training and 20% theoretical study. However, it is unfortunate that students do not receive adequate scopes for practical training [43]. All these facts have contributes to the stagnation of healthcare digitalization in Bangladesh. Therefore, it is necessary to update the curriculum and train the medical teaching staff so that proper training and expertise are provided to the medical students.

#### 4.5.4. Data Security and Privacy

In our study, we found that the majority (73.75%) of the professionals are concerned about the privacy of patient data. Patient health information may include very personal and sensitive information about their life and family. As a result, both the security and the privacy of patient data are very important. Data security protects data against malicious threats, while privacy ensures that data are accessed only by authorized personnel. Digital health enables data to be shared and transferred over the network; it is another challenge to keep data secure during transformation. Blockchain technology can be integrated with the healthcare system to ensure data privacy.

## 5. Discussion

Patents and research publications are important inputs for identifying technological trends and shifts. There has been a significant increase in the number of patent applications [44,45] and publications [46,47,48,49,50,51] related to AI and digital technology-based diagnosis and healthcare applications in recent years. Furthermore, the number of FDA-approved AI/ML-enabled medical devices and applications is increasing significantly [52,53]. This suggests that healthcare digitalization is on the horizon. As a result, we must prepare for the digitalization and speed the transition. In this study, we surveyed different professionals involved in healthcare digitalization and combined their responses to create a picture of their overall perception. This assisted us in comparing their attitudes towards various aspects of digitization, understanding their potential, and identifying the source of stagnation of healthcare digitalization in Bangladesh. We believe that addressing these issues will help digitalization.

The findings of this survey indicate a mixed attitude of professionals, where the majority of the students were highly interested in digital health, and the doctors were pessimistic. However, this study found doctors and pathologists more prepared than students, radiologists and technicians based on their familiarity with digital health literacy. This study also reports the influence of male and private institution professionals on the technical competency and psychological consciousness of digital health.

A large number of votes in Figure 4 showing fear of losing jobs and fear of losing contact with colleagues and patients, and respondents’ reluctance to digitalize in Figure 8 indicate a lack of determination as a major issue that could have contributed to the stagnation of digitalization. Another challenge of going digital is the lack of technical expertise for many professionals in Table 1 and low familiarity scores in Figure 13. Thus, similar to previous studies, this study reports inadequacy of technical skills and determination as challenges to the practical and successful digitalization of healthcare.

A large number of professionals have high psychological competency but lack adequate technical skills, and thus belong to the high–low region of Figure 16. For making digitalization successful, it is important to prepare medical students and other medical professionals accordingly, so that they can easily adapt to digital transformation. The current medical curriculum of Bangladesh lacks sufficient training and education related to digitalization. Therefore, it is necessary to advance the curriculum and develop strategies to prepare medical students as well as to train and motivate other health professionals to make digitalization successful. On the other hand, some professionals have good technical competency but lack confidence, belonging to the low–high region of Figure 16. Consultation, training and empirical demonstration could help them boost their confidence and create a positive outlook toward digital health.

Many professionals voted ‘unwillingness’ as a major challenge and believe that digitalization will only benefit professionals and patients in urban areas. Only proper education and training can correct these misconception. However, health digitization could be a viable solution for improving health services and ensuring health equity. One of the major reasons why rural healthcare is insufficient is a lack of diagnostic and logistical support. Digitalization can help improve this. AI-powered diagnostic pathology and radiology enables accurate diagnosis and disease detection. This will undoubtedly reduce the workload of pathologists and radiologists, thereby improving healthcare in rural and suburban areas.

Digitalization is mostly driven by technological advancement, which is usually costly. Rahimi et al. [54] identified cost as a major challenge to health digitalization. This study also reports cost as a major challenge, as agreed to by 92% (644/701) of respondents. Digital health has the potential to address Bangladesh’s healthcare issues by increasing the reach, impact and efficiency of healthcare services. To accomplish this, however, a sustainable and long-term plan is required, and to drive the digitalization process, economic and technological investments are required. Moreover, the investments should be efficiently coordinated to meet the requirements of health needs. To ensure the practical usability of digital health systems, it is necessary to develop them in collaboration with health professionals by incorporating their feedback.

We also identified some other challenges based on respondent comments, such as the lack of medicolegal guidelines and organizational structure.

## 6. Limitations of This Study

The findings of this study are subject to several limitations, the most important of which is generalizability. Although, we included at least one medical student and doctors from each (37/37) public medical college and hospital of Bangladesh in this study, only 62% (45/72) of the private medical colleges and hospitals were covered. Among the 960 private hospitals and health centers, we reached only 33 (3.4%). No midwives or nurses participated in the survey, yet they play important roles in providing healthcare. Another limitation is that only 22% of the respondents were from outside the city, which included a small number of rural professionals. Non-uniform categorization of the respondents is also a limitation of this study.

This survey was designed to allow health professionals to participate voluntarily, which may have resulted in self-selection bias. Some responses to questions may have influenced the analysis, because options such as “Basically know” and “Neutral” could be interpreted differently by respondents. The question intended to determine whether a respondent had used computers, AI/CAD, or health applications was a ‘Yes/No’ question; this may limit the validity. Some respondents may not have felt encouraged to provide accurate and honest answers, as no incentives were provided to them for participating in this survey.

## 7. Conclusions

The acceptance of digital healthcare among healthcare professionals is crucial for its successful implementation. This study presents important facts that explain health professionals’ perspectives. The impacts of gender, age, location, institution and type of profession on perception was also investigated. Furthermore, we prioritized digitalization challenges and categorized professionals based on their potential. Finally, this study suggested solutions to overcome the challenges for successful digitalization. In the future, we plan a survey to understand the perception of health consumers toward digital health.

## Figures and Tables

**Table 1:** Respondents who have used a computer, digital health applications and/or AI/CAD systems.

Criteria	Count	Profession-Wise Distribution	Location-Wise Distribution	Sector-Wise Distribution
Used 3 technologies	54 (7.7%)	Student: 30 Doctor: 19 Pathologist: 1 Radiologist: 2 Technician: 2	Inside city: 40 Outside city: 14	Public: 31 Private: 23
Used at least 2 technologies	476 (68%)	Student: 134 Doctor: 254 Pathologist: 28 Radiologist: 48 Technician: 12	Inside city: 391 Outside city: 85	Public: 237 Private: 239
Used at least 1 technology	690 (98.5%)	Student: 269 Doctor: 286 Pathologist: 51 Radiologist: 62 Technician: 22	Inside city: 540 Outside city: 150	Public: 353 Private: 337
Used no technologies	11 (1.5%)	Student: 5 Doctor: 0 Pathologist: 2 Radiologist: 2 Technician: 2	Inside city: 6 Outside city: 5	Public: 5 Private: 6

**Table 2:** The influence of different variables on technical competency related to digital health.

Variables	Degrees of Freedom	Critical Value for 5% Level of Significance	Chi-Square Value	*p*-Value	Result
**Age**	12	21.02	82.02	<0.001	Significant relation
**Gender**	8	15.50	2.44	0.965	No relation
**Location**	4	9.48	18.78	0.0008	Significant relation
**Profession**	16	26.29	71.02	<0.001	Significant relation
**Institution**	4	9.48	6.79	0.147	No relation

**Table 3:** The influence of different variables on psychological competency.

Variables	Degrees of Freedom	Critical Value for 5% Level of Significance	Chi-Square Value	*p*-Value	Result
**Age**	12	21.02	40.82	0.00005	Significant relation
**Gender**	8	15.50	11.41	0.179	No relation
**Location**	4	9.48	16.82	0.002	Significant relation
**Profession**	16	26.29	115.02	<0.001	Significant relation
**Institution**	4	9.48	8.62	0.071	No relation

**Table 4:** Frequency of selected keywords in medical curricula and syllabi.

Keywords	The Johns Hopkins University School of Medicine, USA	Medical and Dental Education Curriculum, Bangladesh	All India Institute of Medical Science, India	Medical School of Tokyo University, Japan
**Informatics**	24	2	2	6
**Imaging**	88	12	71	35
**Image**	21	24	9	23
**Digital**	4	7	4	2
**Machine**	18	10	11	4
**Machine Learning**	6	0	0	1
**Computer**	19	28	42	1
**Whole-Slide Image**	0	0	0	0
**Electronic**	15	3	3	3
**Artificial Intelligence**	4	0	1	0
**Total frequency**	**199**	**86**	**143**	**75**

## Data Availability

The Python codes used in the experiment and the survey questionnaire used for this survey can be found at GitHub: https://github.com/shimulshakhawat/Analysis_of_Healthcare_Digitalization_Survey_Data (accessed on 19 October 2022).
